# New Teixobactin Analogues with a Total Lactam Ring

**DOI:** 10.1021/acsmedchemlett.3c00435

**Published:** 2023-11-14

**Authors:** Giuseppe Scioli, Lorenza Marinaccio, Marta Bauer, Wojciech Kamysz, Anish Parmar, Enas Newire, Ishwar Singh, Azzurra Stefanucci, Adriano Mollica

**Affiliations:** † Department of Pharmacy, University “G. d’Annunzio” Chieti-Pescara, Via dei Vestini 31, 66100 Chieti, Italy; ‡ Department of Inorganic Chemistry, Faculty of Pharmacy, Medical University of Gdańsk, 80-416 Gdańsk, Poland; § Antimicrobial Pharmacodynamics and Therapeutics, Department of Molecular and Clinical Pharmacology, University of Liverpool, Sherrington Building, L69 3GA Liverpool, U.K.; ∥ Department of Chemistry, The Robert Robinson Laboratories, The University of Liverpool, L69 3BX Liverpool, United Kingdom

**Keywords:** Teixobactin, Cyclic peptide, Solid phase peptide synthesis, SPPS, Resin, Resistance, Bacteria

## Abstract

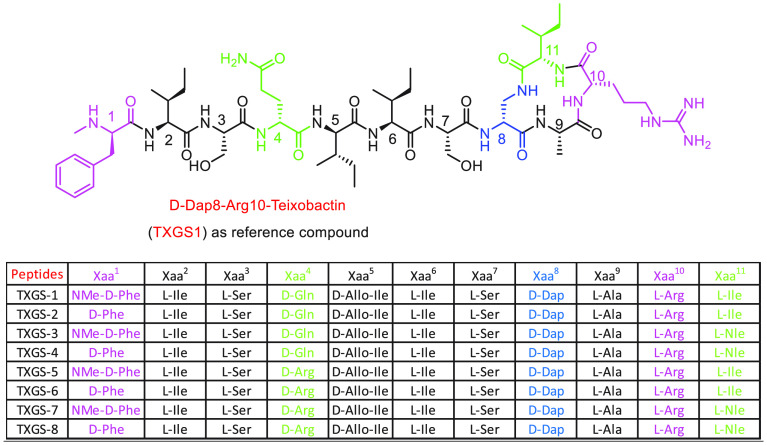

Teixobactin is a new antibiotic peptide with strong efficacy against several Gram-positive resistant bacteria, the structure of which is extremely difficult to obtain in the laboratory via multistep conventional synthesis. To face the increasing antibiotic resistant bacteria, it is fundamental to introduce new types of antibiotics with innovative mechanisms of action without resistance; thus, many scientists are studying and developing new methods to synthesize teixobactin analogues. In this work, seven Arg_10_-teixobactin analogues with a total lactam ring have been prepared via solid phase peptide synthesis. In order to obtain the total lactam ring, d-Thr_8_ was replaced by (2*R*,3*S*)-diamino-propionic acid. To verify their antimicrobial activity and efficacy, each analogue was tested with MIC against different resistant pathogens, showing an interesting activity for Nle^11^ containing compounds.

Teixobactin is an antibiotic peptide isolated by Ling et al. in 2015 from the noncultivable bacterium *Eleftheria Terrae*.^1,2^ This new molecule has attracted the attention of the scientific community thanks to its high antimicrobial activity against several resistant Gram-positive bacteria, which are difficult to treat with the most common antibiotics (*e.g. Staphylococcus aureus*, MRSA, and *Mycobacterium tuberculosis*), and against *Clostridium difficile* and *Bacillus anthracis*.^1^ It acts on Gram-positive bacteria in such a way that it is difficult for them to become resistant to it. Due to the fact that the target of the antibiotics is not easy to modify by the bacteria, the resistance mechanism would take a much longer time to develop. This molecule is effective against MRSA; thus, it could be used to fight against antibiotic-resistant strains. From a structural point of view, teixobactin is a *head to side chain* macrocyclic depsipeptide of 11 residues; among them four are d-amino acids. Six of them possess hydrophobic side chains, along with one rare amino acid called l-*allo*-enduracididine; its limited availability is a hindrance in the development of teixobactin analogues because the synthetic preparation is very tedious and challenging.^3^ Thus, most of the initial efforts have been focused on its replacement with natural and readily accessible amino acids. l-*allo*-Enduracididine’s charged side chain is important for the antimicrobial efficacy, but it can be substituted with arginine and lysine without significant loss of activity;^4^ since arginine is readily available in nature, it was considered as a surrogate of enduracidinine in the analogous design and structure activity relationship studies of teixobactin (Figure 1).^4−6^

**Figure 1:**
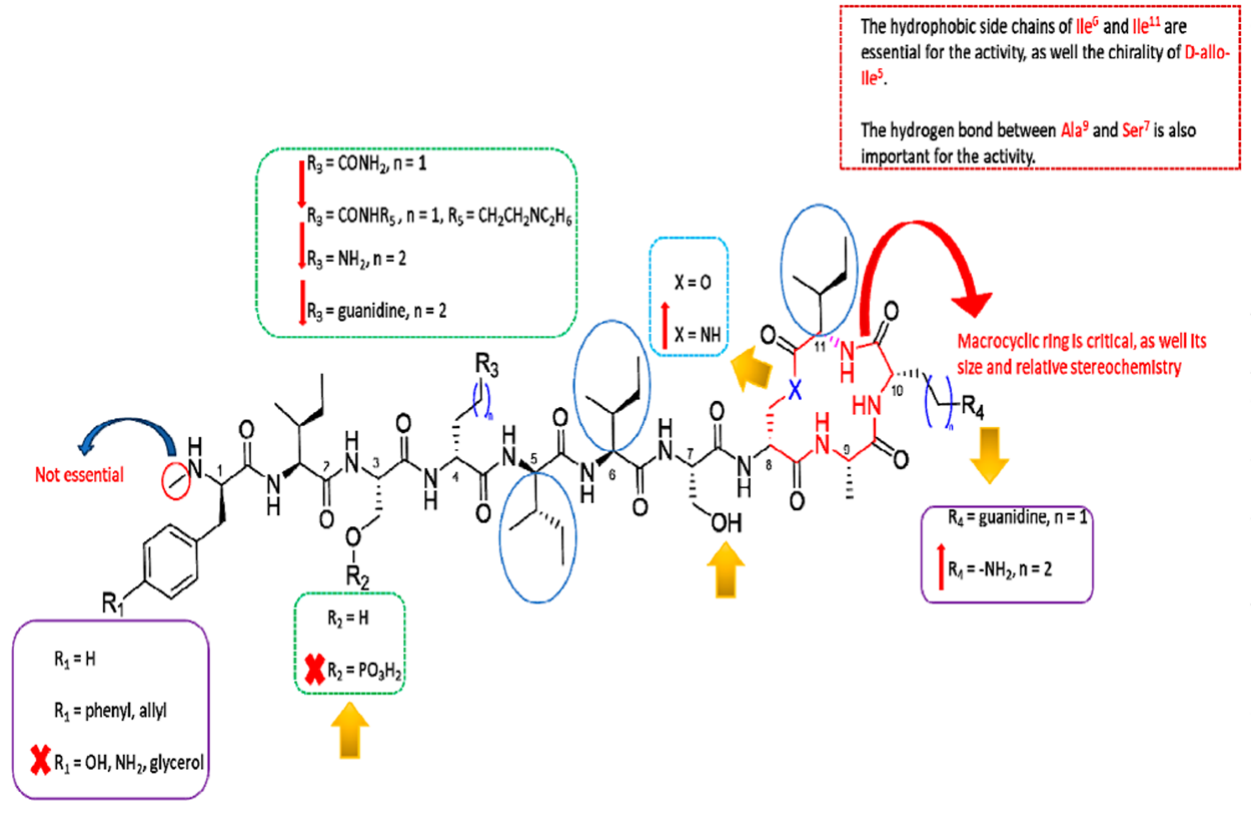
Representation of structure–activity relationships for teixobactin.

The *N*-terminus of teixobactin contains the unnatural amino acid methyl-d-phenylalanine; any change in stereochemistry causes a complete loss of activity, while the methyl group is not essential. Increasing the hydrophobicity of the d-phenylalanine side chain improves the potency of new analogues. Two serine residues at positions 3 and 7 exert diverse structural roles; the crystal structure analysis reported by Yang et al. on a truncated analogue of teixobactin described the presence of a hydrogen bond interaction between the NH group of Ala_9_ and the side chain of Ser_7_.^7^

The replacement of Ser_7_ with alanine induces a loss of activity; thus, these two residues cannot be substituted. Otherwise Ser_3_ is prone to modification.^8^ A noncharged polar d-glutamine is located in position 4, and its stereochemistry is crucial to guarantee the activity; its replacement with other noncharged or charged residues causes a drop in activity, while the combination of this modification with hydrophobic substitution at the *N*-terminus potentiates the membrane anchoring capacity, ultimately leading to improved activity of teixobactin analogues.^9^ Four isoleucines are at position 2, 5, 6, and 11, and one of them is an unnatural residue; it was reported that their substitution gives inactive or very poorly active analogues. In particular double substitution of Ile_6,7_ is detrimental for bioactivity.^10^ Replacement of Ile_11_ with Nle induces a slight enhancement in efficacy due to the reinforcement of hydrophobic interactions.^3^ The macrocyclic ring is essential for biological activity, being involved in hydrogen bonding with the lipid II pyrophosphate group and cell wall precursors.^11^ Replacement of the lactone group with a lactam moiety through the insertion of d-diamino-propionic acid in place on d-Thr_8_ results in an analogue more potent than teixobactin, supporting the hypothesis that an additional amide group increases the binding affinity for lipid II.^12,13^ It was demonstrated that the importance of the macrocyclic moiety resides in the ability of the amide groups of Ser_7_, Arg_10_, Ile_11_, and the guanidine group to form a cavity able to bind a chloride ion.^13^ Furthermore, teixobactin interacts as β-sheet dimer with cell wall membrane components, thus generating two cavities comprising the *C*-terminal cycle and *N*-terminus acting as receptor for pyrophosphate groups via hydrogen bonding.^13^ As observed by the X-ray crystallographic structure of teixobactin analogues, a lactam bridge in place of a lactone may improve the interaction with lipids II and III; however, ring expansion resulted in analogues with very poor activity.^12,13^ The solid state NMR work reported by Shukla and co-workers helps to clarify the importance of the relative stereochemistry and structural features of teixobactin: The use of a labeled analogue reveals the ability of this molecule to form a large cluster on the membrane surface by oligomerization of lipid II-binding teixobactin.^11−14^ This molecule assumes a β-sheet form in which the critical sequence of d- and l-amino acids allows separation of hydrophobic and hydrophilic side chains located at the same side of the β-sheet, a common behavior of diverse naturally occurring peptides.^15−17^ The peptide head containing ring interacts with *N*-acetyl muramic acid and to a minor extent with *N*-acetyl-glucosamine; the tail is anchored on the membrane surface by two isoleucines. In this way teixobactin significantly perturbs the bacterial membrane lipids and cell wall biosynthesis.^11,18^

Taking into consideration all these data, we planned to prepare a series of novel depsipeptide analogues of teixobactin, in order to simplify the synthetic protocol and to solve some physicochemical limitations which preclude a good overall yield and an easy isolation of pure peptide from the crude mixture.^19−25^ Ultimately we also aimed to expand the spectrum of activity against a large panel of bacteria in comparison to that previously observed for teixobactin and its analogue d-Dap_8_,Arg_10_-teixobactin.^24,7^ Based on these SAR studies, we have synthesized the *lead compound*d-Dap_8_,Arg_10_-teixobactin (**TXGS-1**) as reference and seven new teixobactin analogues containing (2*R*,3*S*)-diamino-propionic acid in place of the d-Thr_8_ in order to obtain a total lactam ring (**TXGS-2–8**). In our design, in which the first residue has been maintained or substituted with d-phenylalanine, both d-glutamine and d-arginine are located in position four, and l-isoleucine or nor-leucine has been placed in position 11 with the aim to explore the influence of an additional charged and hydrophobic residue on the antimicrobial activity of the novel peptides. Due to the strict requirements of the pharmacophoric motif, the lactam ring has been retained in line with the reference compound **TXGS-1** (Figure 2).^7^

**Figure 2:**
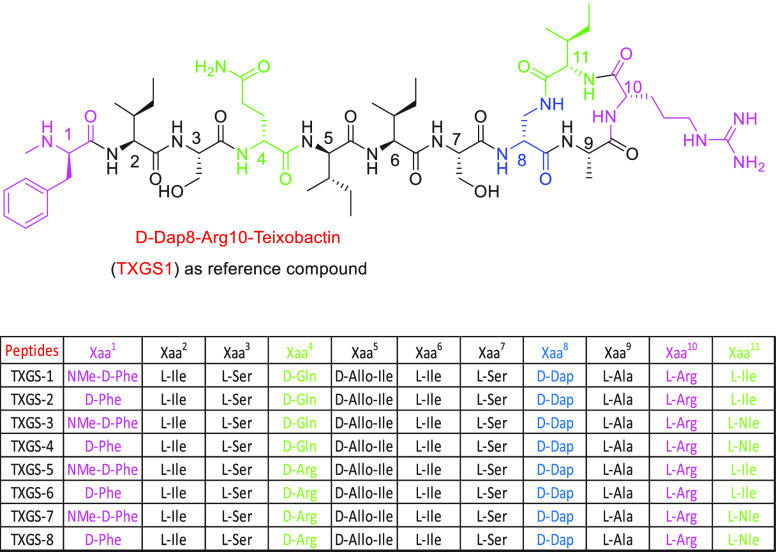
Design of the novel depsipeptide analogues of teixobactin starting from the *lead compound***TXGS-1**.

A total solid phase peptide synthesis has been developed to reach the linear fully protected sequence, and then a soft cleavage was applied to the resin-bound peptide in order to remove the sole protecting group of the d-amino propionic acid (Scheme 1). The crude linear peptide has been submitted to the cyclization reaction in solution at high dilution condition and then fully deprotected with a mixture of TFA/TIS/water to afford the desired depsipeptide as both *N*-methylated (**TXGS-1,3,5,7**) or des-methylated (**TXGS-2,4,6,8**) analogue (Scheme 1). It is worth noting that in order to obtain the right cycle in the final molecular structure, the cyclization reaction should occur between the *C*-terminus of the peptide and the selectively deprotected -NH_2_ group of the lateral chain of d-Dap_8_. A total removal of protecting groups cannot be done because many different collateral cyclization reactions can occur.^25,26^ For this reason, we have chosen a d-Dap protected with a methyltrithyl group (Mtt): this protecting group can be removed using a 1% TFA solution, which allows cleavage of the peptide sequence from 2-CT-Cl resin without removal of the other side chain protecting groups. After completing the peptide’s elongation, a soft cleavage with a solution of 1% TFA in DCM was added to the resin to release the sole linear precursor with a *C*-terminus and -NH_2_ lateral chain of d-Dap free to react. The last amino acid (e.g., d-Phe or N-Me-Phe) was protected with the Boc group to perform the final total deprotection.

**Scheme 1:**
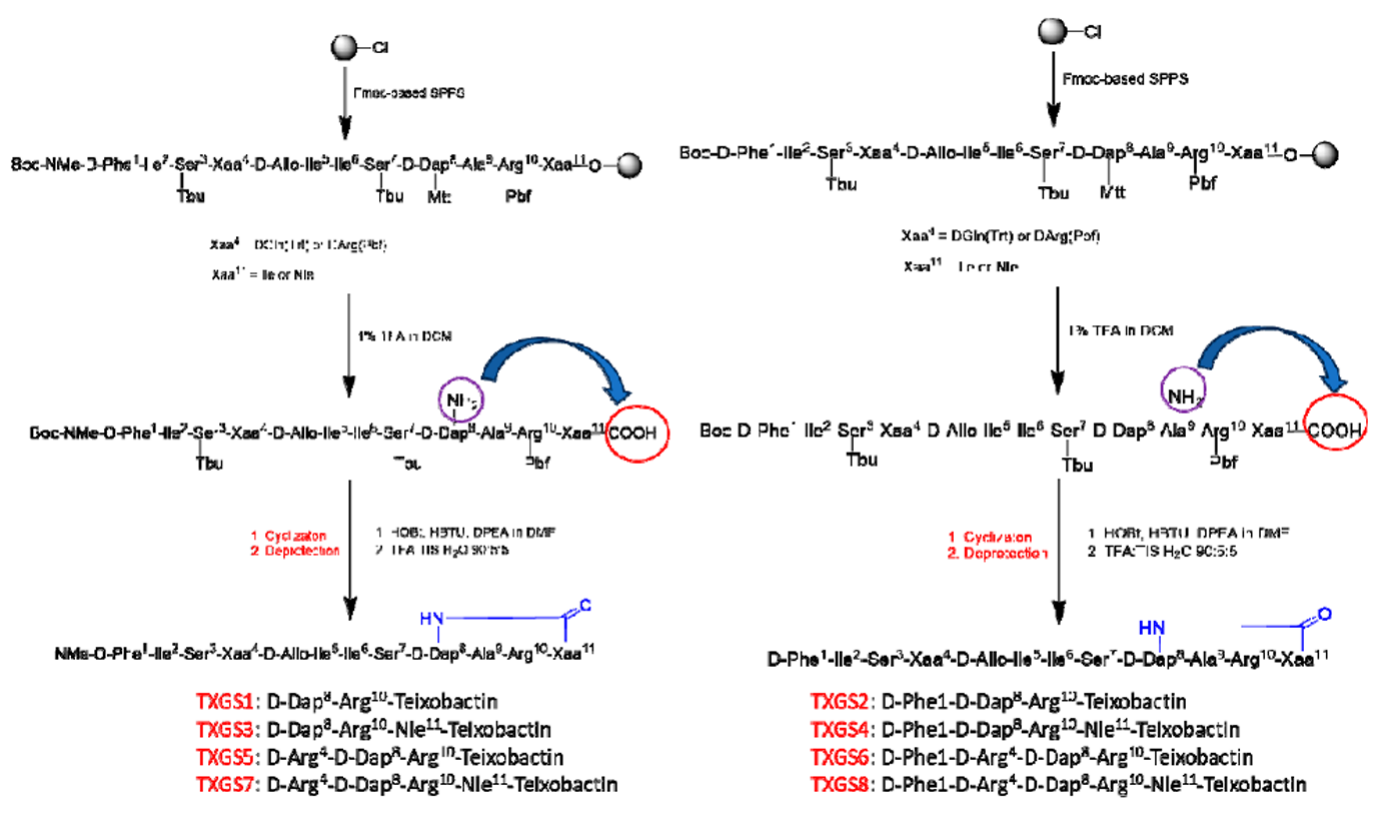
Synthetic Strategy Applied to Prepare Teixobactin’s Analogues

All crude peptides were purified by RP-HPLC, and the overall yields were calculated after that (Table S1, see SI). Then the purity of the isolated peptides was checked by analytical RP-HPLC and confirmed to be ≥95%, LRMS data were collected for each pure peptide to check their molecular identity (see SI). The antimicrobial activity evaluation was performed using bacterial strains readily available in the laboratory and vancomycin and ketoconazole as conventional antibiotic and antifungal agents for reference (Table 1).^27,28^

**Table 1:** MIC Values of New Teixobactin Analogues^a^

peptides	*MRSA* ATCC 33591	*S. aureus* ATCC 25923	*E. coli* ATCC 25922	*S. epidermidis ATCC 14990*	*C. glabrata* ATCC 15126
2TXGS-1*	ND	32	>512	256	>512
**TXGS-2**	ND	32	>512	128	>512
**TSGX-3**	4	4	>512	64	>512
**TXGS-4**	4	4	>512	8	>512
**TXGS-5**	ND	256	256	256	512
**TXGS-6**	ND	64	>512	64	>512
**TXGS-7**	4	4	32	8	64
**TXGS-8**	ND	8	64	8	128
**Vancomicin**	16	2	>64	2	-
**Ketoconazole**	-	-	-	-	0.62

a **TXGS-1** is the analogue used as *lead compound*. ND = not determined.

To determine the antimicrobial activity of these analogues, the MIC test using the microdilution method was chosen. Analogues were tested on three different types of bacteria: Gram-positive *S. aureus* and *S. epidermidis*, Gram-negative *E. coli* and fungus *C. glabrata* (Table 1) including the MRSA ATCC 33591. Interestingly, **TXGS-3,4,7** showed similar activity when compared to *S. aureus* ATCC 25923 against the methicillin-resistant *S. aureus* ATCC 33591; however, these are more potent than vancomycin. The substitution of d-Gln in position 4 with d-Arg did not show any important change in antimicrobial activity as well as the presence of a methyl group at the *N*-terminus. To our surprise, an improved activity against all of them was observed for **TXGS-3,4,7,8** on *S. aureus* in comparison with the reference peptide **TXGS-1** as control. Peptides **TXGS-7,8** are more effective than the *lead compound* against *E. coli* and *C. glabrata*. These sequences possess a residue of Nle_11_ in common which seems to be responsible for the enhanced antimicrobial activity *in vitro*, while the presence of the other amino acids is not discriminant in this sense. Unfortunately, none of them seem to be more potent than the *lead compound* against *S. epidermidis*. These analogues present the same potency (4 μg/mL), and they show reduced antimicrobial activity compared to natural teixobactin (0.25 μg/mL).^29,30^ Notably the presence of d-Arg_4_ in **TXGS-7**, d-Phe_1_ in **TXGS-4**, and Nle_11_ in all the tested compounds is not discriminant for their antimicrobial activity against MRSA, as well as the presence of two/three cationic charges. Seven new teixobactin analogues were synthesized using a tandem solid phase peptide synthesis (SPPS)/solution cyclization strategy. The Mtt-protected d-Dap allows a selective cyclization reaction without involving other functional groups in the peptide’s linear sequence, thus furnishing an efficient method to obtain total lactam ring teixobactin analogues. Even if our strategy supports the SPPS as a straightforward technique to readily prepare teixobactin analogues in the laboratory, some solubility limitations still exist for some of them, which render the overall yields extremely low. This preliminary study helped us to reach an easy but efficient synthetic protocol via SPPS which overcomes drawbacks related to the original teixobactin synthesis and those of some described analogues.^4,29−31^ Depsipeptides **TXGS-3,4,7** show good antimicrobial activity against a broad panel of bacteria and moderate potency against MRSA. Further work is necessary to solve solubility problems during HPLC purification, to delineate a complete bioactivity profile against a large panel of pathogens, and to prove the efficacy *in vivo*.

## Experimental Section

### Materials and Chemicals

All reagents were purchased and used without any further purification or treatment. 2-CT-Cl resin with a loading factor of 1.06 mequiv/g (100/200 mesh) was purchased by Novabiochem. Fmoc-Nle-OH, Fmoc-d-Dap(Mtt)-OH, Fmoc-d-Gln(Trt)-OH, Fmoc-d-allo-Ile-OH, Fmoc-d-Arg(Pbf)-OH, and N-Me-Boc-d-Phe-OH were purchased by Iris Biotech, while Fmoc-Ile-OH, Fmoc-Ala-OH, Fmoc-Arg(Pbf)-OH, DIPEA, and HOBt were purchased by GL Biochem (Shangai) Ltd. Finally, HBTU, Fmoc-Ser(Otrt)-OH, and Boc-d-Phe-OH were purchased by Fluka. Solvents DMF, CH_3_OH, DCM, diethyl ether, acetonitrile, TIPS, and piperidine were from Merck (Sigma-Aldrich). Syringes were purchased from Torviq, Canada. Crude peptides were purified with a semipreparative RP-HPLC Waters column with a Luna C18(2), 5 μm 100 Å, 250 × 10 mm semipreparative chromatographic column (4 mL/min), gradient of ACN:H_2_O (5:95), and time course of 30 min. Fractions were collected and lyophilized with a Lyovapor L-200 BUCHI lyophilizer. Analytical HPLC analysis was performed with a XBridge C18, 5 μm, 250 × 4.6 mm column (1 mL/min), gradient of ACN:H_2_O (5:95), and time course of 24 min. ^1^H NMR spectra were recorded at 25 °C with a 300 MHz Varian Oxford spectrometer, DMSO-*d*_6_ as solvent, and chemical shifts in parts per million (δ) downfield from TMS. No unexpected or unusually high safety hazards were encountered.

### Synthesis

The standard SPPS procedure was followed, using 3 equiv of each amino acid for each coupling and HBTU (3 equiv), HOBt (3 equiv), and DIPEA (6 equiv) dissolved in 4 mL of DMF as the coupling mixture.^15,16^ 120 mg of 2-CT-Cl resin (1 equiv) was weighed into a syringe for manual solid phase synthesis and swelled in DCM (8 mL) for 1 h with an automatic shaker. For the first coupling, 5 mL of a DCM solution containing the first amino acid and DIPEA was added to the resin and shaken overnight. The day after, the capping procedure was applied using 20 mL of a mixture of DCM:CH_3_OH:DIPEA (85:10:5) (three times, 15 min each). Then the resin was washed with DMF (3×), CH_3_OH (3×), and DCM (3×), and 8 mL of a solution of piperidine/DMF 20% v/v was added to resin and the mixture was shaken for 15 min. This procedure was repeated two times for each protected amino acid. After Fmoc-deprotection, the resin was washed with DMF (3×), CH_3_OH (3×), and DCM (3×), and the corresponding amino acid coupling mixture was added. After 2 h the resin was washed with DMF (3×), CH_3_OH (3×), and DCM (3×), and the Kaiser test on a small amount of resin was done to confirm the correct occurrence of coupling. Then soft cleavage was done using 4 mL of a solution of TFA/DCM 1% v/v with shaking for 3 h. The solution was transferred into a 100 mL round-bottom flask and TFA, was removed with DCM in a rotary evaporator. The crude product was precipitated with ice-cold diethyl ether using a centrifuge at 4400 rpm for 3 min (this procedure was repeated five times). The supernatant was transferred into a plan flask, and the white powder was dried at high vacuum for 3 h. The cyclization step was performed by dissolving HOBt (6 equiv), HBTU (6 equiv), and DIPEA (6 equiv) in 175 mL of DMF in a 500 mL round-bottom flask, while the linear precursor was dissolved into 25 mL of DMF and added dropwise with a loading funnel. The reaction mixture was stirred overnight. Then the solvent was removed by a rotary evaporator and the crude powder was dried under high vacuum for 2 h. The final total deprotection was performed using 15 mL of a mixture of TFA:TIS:H_2_O (90:5:5) in a reaction flask with stirring for 3 h. TFA was removed with DCM using a rotary evaporator, and the remaining solution was put into four vials containing ice-cold diethyl ether to allow the precipitation of the crude product with a centrifuge at 4400 rpm for 3 min. The supernatant was transferred into a plan flask, and the white powder was dried at high vacuum for 4 h.

### HPLC Analysis

To evaluate the presence of the linear precursor, 1 mg of the white powder obtained after SPPS and soft cleavage of each crude linear peptide was dissolved in 1 mL of CH_3_OH; 200 μL of this sample was injected in a semipreparative column Luna C18(2), 5 μm 100 Å, 250 × 10 mm, with a flow of 4 mL/min using a gradient of ACN:H_2_O and a 30 min time course. The chromatographic peak of each linear sequence has a retention time in the range of 20–22 min, with high intensity (254 nm wavelength) and straight shape. The same analysis was applied to evaluate the completeness of the cyclization reaction. The purification of the crude depsipeptide was performed with a semipreparative column at a flow of 4 mL/min, a gradient of ACN:H_2_O, and a 30 min time course. Samples were prepared by dissolving 10 mg of product into 1 mL of a mixture of ACN:H_2_O 1:1, and 500 μL was injected. Each fraction has been checked with LRMS and collected in a round-bottom flask, evaporated into a rotary evaporator, and lyophilized overnight. Analytical HPLC was performed with an XBridge C18, 5 μm, 250 × 4.6 mm column, at a flow of 1 mL/min. All samples were prepared by dissolving 1 mg of product in 1 mL of CH_3_OH, injection volume of 20 μL, gradient of ACN:H_2_O, and 24 min time course. Chromatographic peaks corresponding to the final products have a retention time in the range 19–22 min (see SI).

### Mass Spectra

Mass spectra were performed on an LCQ (Finnigan-Mat) ion trap mass spectrometer (San Jose, CA, USA) equipped with an electrospray ionization source. The capillary temperature was set at 300 °C, and the spray voltage was set at 3.5 kV. The fluid was nebulized by using nitrogen as both the sheath and the auxiliary gas. A sample of 1 mg/mL of the pure lyophilized peptide in methanol for mass spectroscopy was injected into the apparatus in a volume of 0.01 mL. Results of the mass spectra are expressed as the *m*/*z* ratio.

### Antimicrobial assays

. The minimum inhibitory concentrations (MICs) of synthesized compounds were assessed according to the broth microdilution method using 96-well plates, in reference to the protocol of the Clinical and Laboratory Standards Institute (CLSI).^32,33^ In assays, reference strains of the bacteria *S. aureus* ATCC 25923, *S. epidermidis* ATCC 14990, and *E. coli* ATCC 25922 and the fungus *C. glabrata* ATCC 15126 were used, all from The Polish Collection of Microorganism, Polish Academy of Sciences, Wrocław, Poland. Bacteria at initial inoculums of 0.5  ×  105 CFU/mL in Mueller–Hinton Broth (MHB), and fungus at initial inoculums of 2  ×  103 CFU/mL in RPMI-1640, were exposed to the serial dilution of compounds. Tested concentrations were in the range 1–512 μg/mL. 96-well plates with microorganisms and tested substances were incubated at 37 °C for 18 h for bacteria and for 24 h for *C. glabrata*. The MIC values were taken as the lowest concentrations at which visible growth of microorganisms was inhibited. The minimum inhibitory concentrations (MICs) for MRSA ATCC 33591 were tested at the University of Liverpool. Bacterial cultures were grown overnight in Mueller–Hinton Agar (MHA) plates and adjusted to a final concentration of 105–106 CFU/mL. 100 μL of inoculum in Meuller–Hinton broth (MHB) was mixed with an equal volume of peptides (dissolved in MHB) at 2× their concentration in a 96-well plate. In parallel experiments, MIC values were determined in the media containing polysorbate 80 (0.002%, v/v) to prevent nonspecific adsorption of the peptides to plastic surfaces. The final peptide concentrations ranged from 0.0625–32 μg/mL (the lower range 0.031–16 μg/mL was used). Positive and negative controls contained 200 μL of inoculum without any peptide dissolved in broth, respectively. The 96-well plates were then incubated at 37 °C for 24 h. All the experiments were performed in two independent duplicates, and the MIC was determined as the lowest concentration in which no visible growth was observed. The minimum bactericidal concentration (MBC) was determined by plating out the dilution representing the MIC and concentrations up to 16× MIC on MHA plates kept at 37 °C for 24 h.

## Supporting Information Available

RP-HPLC analytical traces and mass spectra (PDF)

## Abbreviations

ACN: Acetonitrile
CH_3_OH: Methanol
DCM: Dichloromethane
DIPEA: Dimethyl–ethyl amine
DMF: N,N-Dimethylformamide
HBTU: 3-[Bis(diethylamino)methyliumyl]-3H-benzotriazol-1-oxide hexafluorophosphate
HOBt: Hydroxy benzotriazole
TIS: Tri-isopropyl silane
TFA: Trifluoracetic acid
2-CT-Cl: 2-Chlorotritilic chloride resin
DMSO: dimethyl sulfoxide
Mtt: Methyltrityl group
HPLC: High performance liquid chromatography
LRMS: Low resolution mass spectroscopy
MIC: Minimum inhibitory concentrations
CLSI: Clinical and Laboratory Standards Institute
MHB: Mueller Hinton Broth
CFU: Colony-forming unit
MHA: Mueller-Hinton Agar.
