# Supplementary material for: New Teixobactin Analogues with a Total Lactam Ring
Source: ACS Med Chem Lett. 2023 Nov 14;14(12):1827–32. doi: 10.1021/acsmedchemlett.3c00435 (PMC10726481; doi:10.1021/acsmedchemlett.3c00435)
Supplement: Supplementary file 1 — ml3c00435_si_001.pdf [file ml3c00435_si_001.pdf]

SUPPORTING INFORMATION

NEW TEIXOBACTIN ANALOGUES WITH TOTAL LACTAM RING

Giuseppe Scioli,[1] Lorenza Marinaccio,[1] Marta Bauer,[2] Wojciech Kamysz,[2] Anish Parmar,[3,4] Enas Newire,[3,4] Ishwar Singh,[3,4] Azzurra Stefanucci,[1,*] Adriano Mollica[1]

[1] Department of Pharmacy, University "G. d'Annunzio" Chieti-Pescara, Via dei Vestini 31, 66100, Chieti, Italy.

[2] Department of Inorganic Chemistry, Faculty of Pharmacy, Medical University of Gdańsk, 80-416 Gdańsk, Poland.

[3] Antimicrobial Pharmacodynamics and Therapeutics, Department of Molecular and Clinical Pharmacology, Universi-ty of Liverpool, Sherrington Building, L69 3GA Liverpool, UK.

[4] Department of Chemistry, The Robert Robinson Laboratories, The University of Liverpool, L69 3BX, Liverpool, United Kingdom.

*corresponding author email: a.stefanucci@unich.it

Table of contents	pages
Analytical data and yields for peptides **TXGS1-8**	S2
HPLC traces	S3,S4
LRMS spectra	S5-S9

Table S1. Analytical data and yields for peptides **TXGS1-8**

Products	Molecular formula	Expected mass (e.m.)	Mass found [M]	Half mass [M/2]	Retention time (min)	Overall yield (%)
**TXGS-1**	$C_{57}H_{96}N_{16}O_{14}$	1229.49	1229.8	615.5	20.69	11.8
**TXGS-2**	$C_{56}H_{94}N_{16}O_{14}$	1215.47	1215.6	-	22.24	8.2
**TXGS-3**	$C_{57}H_{96}N_{16}O_{14}$	1229.49	1229.8	615.5	19.95	6.4
**TXGS-4**	$C_{56}H_{94}N_{16}O_{14}$	1215.47	1215.1	607.7	20.17	5.9
**TXGS-5**	$C_{58}H_{100}N_{18}O_{13}$	1257.55	1257.8	629.7	21.64	4.9
**TXGS-6**	$C_{57}H_{98}N_{18}O_{13}$	1243.52	1243.6	-	19.69	23.1
**TXGS-7**	$C_{58}H_{100}N_{18}O_{13}$	1257.55	1257.8	-	20.64	3.9
**TXGS-8**	$C_{57}H_{98}N_{18}O_{13}$	1243.52	1243.6	622.4	21.88	6.7

Figure S1. HPLC traces of the novel peptides.

TXGS-1: D-Dap_8-Arg_{10}-teixobactin

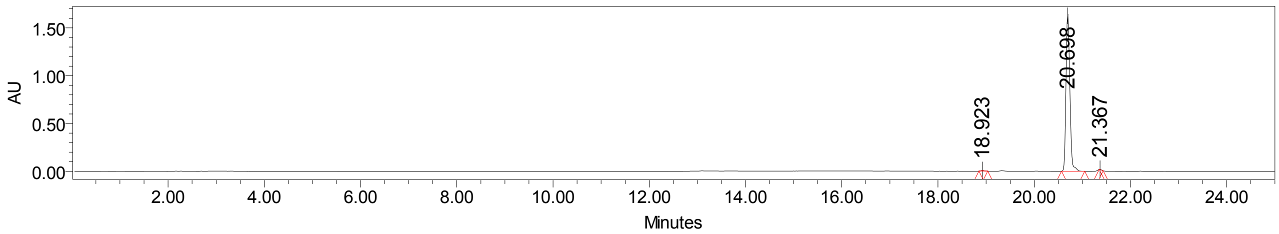

	Name	Retention Time	Area	% Area
1		18.923	34822	0.38
2		20.698	9078516	99.52
3		21.367	8744	0.10

TXGS-2: D-Phe_1-D-Dap_8-Arg_{10}-teixobactin

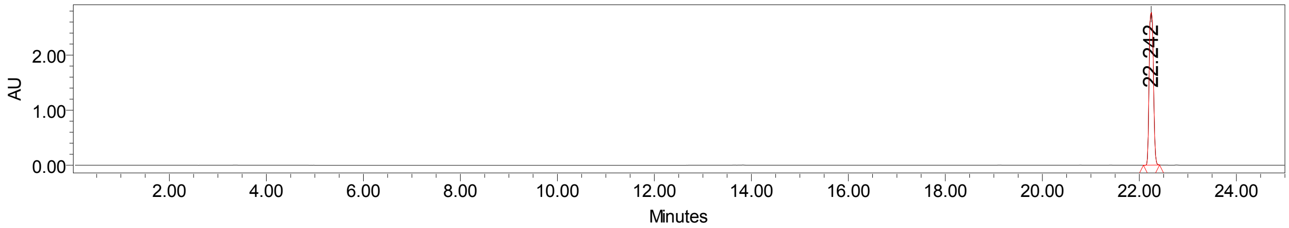

	Name	Retention Time	Area	% Area
1		22.242	4051512	100.00

TXGS-3: D-Dap_8-Arg_{10}-Nle_{11}-teixobactin

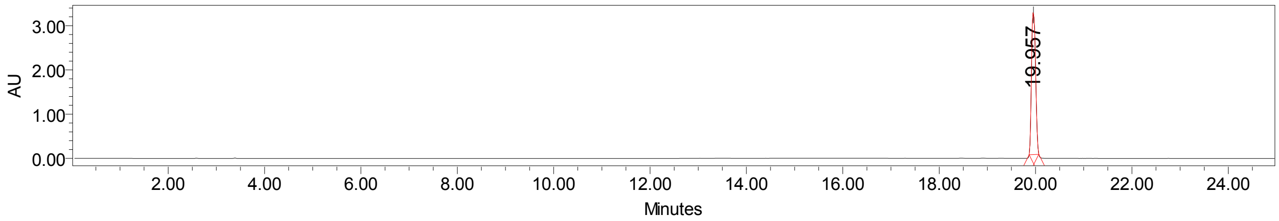

	Name	Retention Time	Area	% Area
1		19.957	18324281	100.00

TXGS-4: D-Phe_1-D-Dap_8-Arg_{10}-Nle_{11}-teixobactin

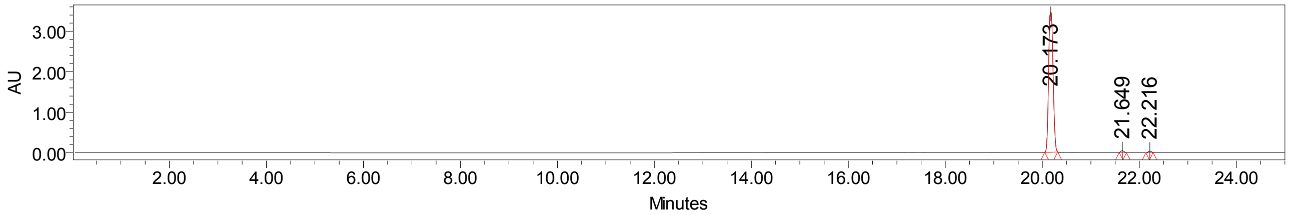

	Name	Retention Time	Area	% Area
1		20.173	21773893	98.27
2		21.649	212193	0.96
3		22.216	170227	0.77

TXGS-5: D-Arg$_4$- D-Dap$_8$-Arg$_{10}$-teixobactin

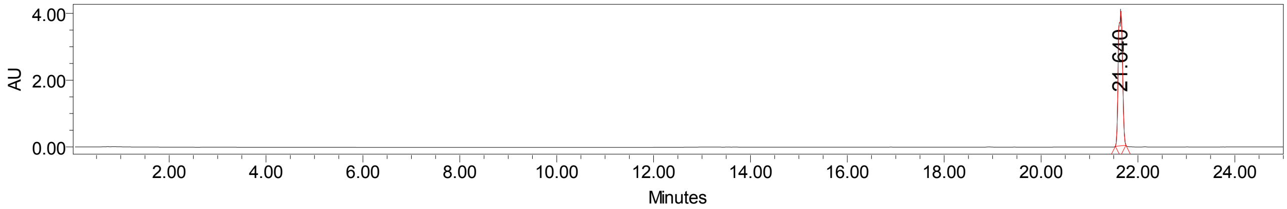

	Name	Retention Time	Area	% Area
1		21.640	24480972	100.00

TXGS-6: D-Phe$_1$-D-Arg$_4$- D-Dap$_8$-Arg$_{10}$-teixobactin

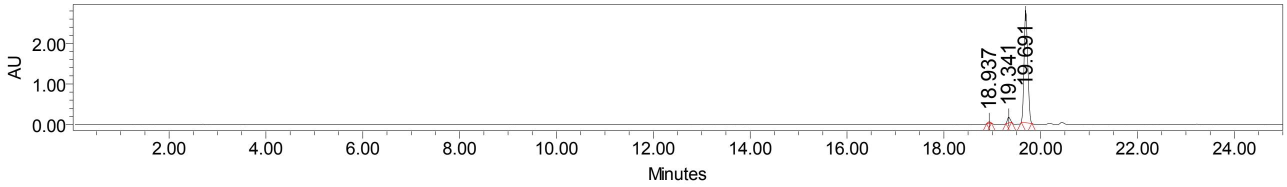

	Name	Retention Time	Area	% Area	Height
1		18.937	138970	0.91	39420
2		19.341	540205	3.52	137881
3		19.691	14646581	95.57	2767830

TXGS-7: D-Arg$_4$- D-Dap$_8$-Arg$_{10}$-Nle$_{11}$-teixobactin

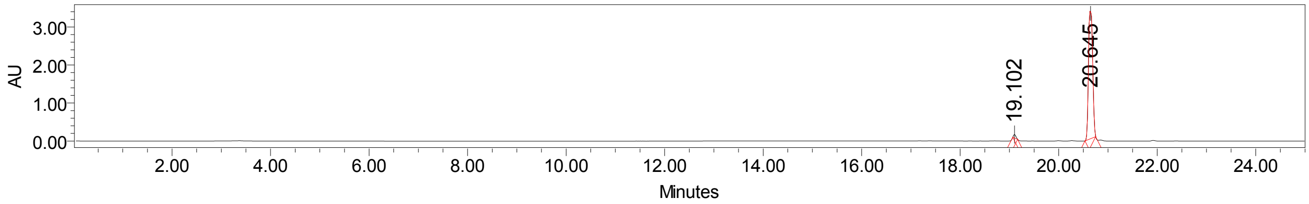

	Name	Retention Time	Area	% Area
2		20.645	20049909	98.44
1		19.102	318404	1.56

TXGS-8: D-Phe$_1$-D-Arg$_4$- D-Dap$_8$-Arg$_{10}$-Nle$_{11}$-teixobactin

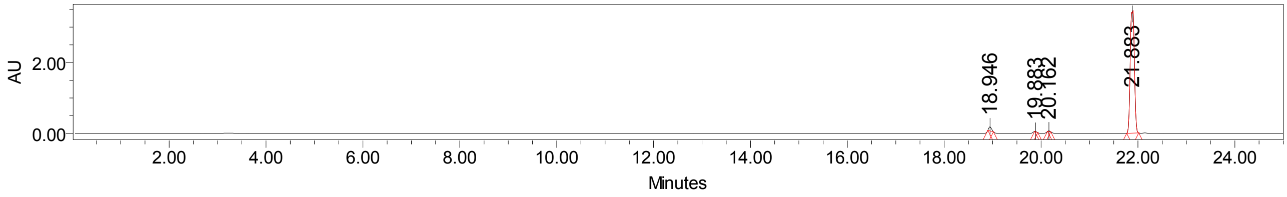

	Name	Retention Time	Area	% Area
4		21.883	20357622	97.43
3		20.162	52166	0.25
2		19.883	19552	0.09
1		18.946	465452	2.23

Figure S2. LRMS of the novel peptides.

TXGS-1

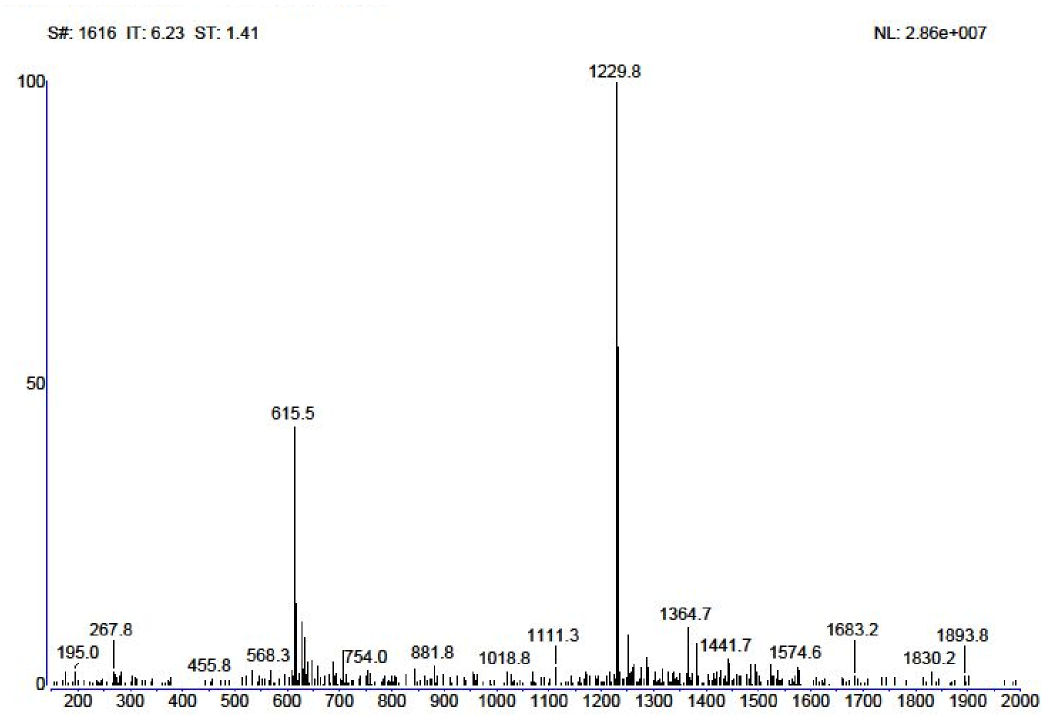

TXGS-2

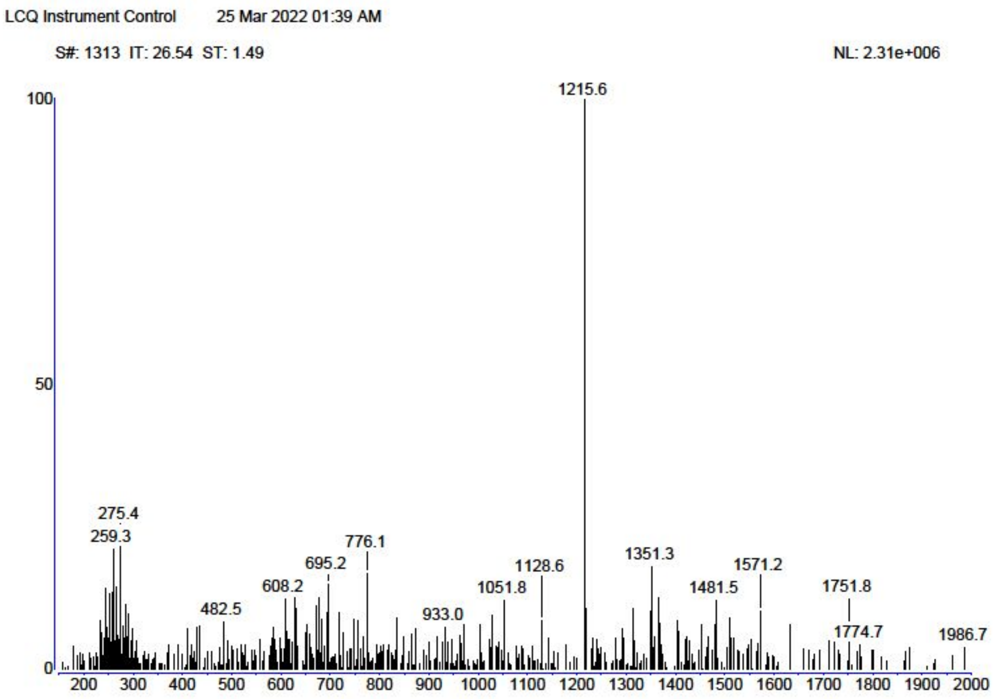
LCQ Instrument Control 25 Mar 2022 01:39 AM
S#: 1313 IT: 26.54 ST: 1.49
NL: 2.31e+006
1215.6
275.4
259.3
776.1
695.2
608.2
482.5
933.0
1051.8
1128.6
1351.3
1481.5
1571.2
1751.8
1774.7
1986.7
100
50
0
200 300 400 500 600 700 800 900 1000 1100 1200 1300 1400 1500 1600 1700 1800 1900 2000

TXGS-3

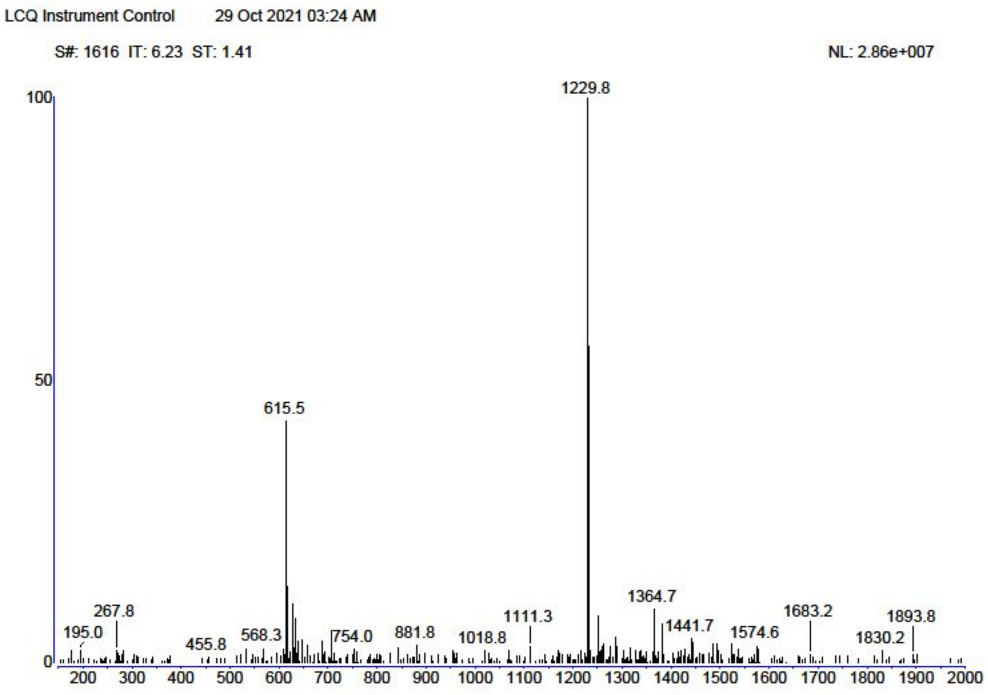
LCQ Instrument Control 29 Oct 2021 03:24 AM
S#: 1616 IT: 6.23 ST: 1.41
NL: 2.86e+007
1229.8
615.5
195.0
267.8
455.8
568.3
754.0
881.8
1018.8
1111.3
1364.7
1441.7
1574.6
1683.2
1830.2
1893.8
100
50
0
200 300 400 500 600 700 800 900 1000 1100 1200 1300 1400 1500 1600 1700 1800 1900 2000

TXGS-4

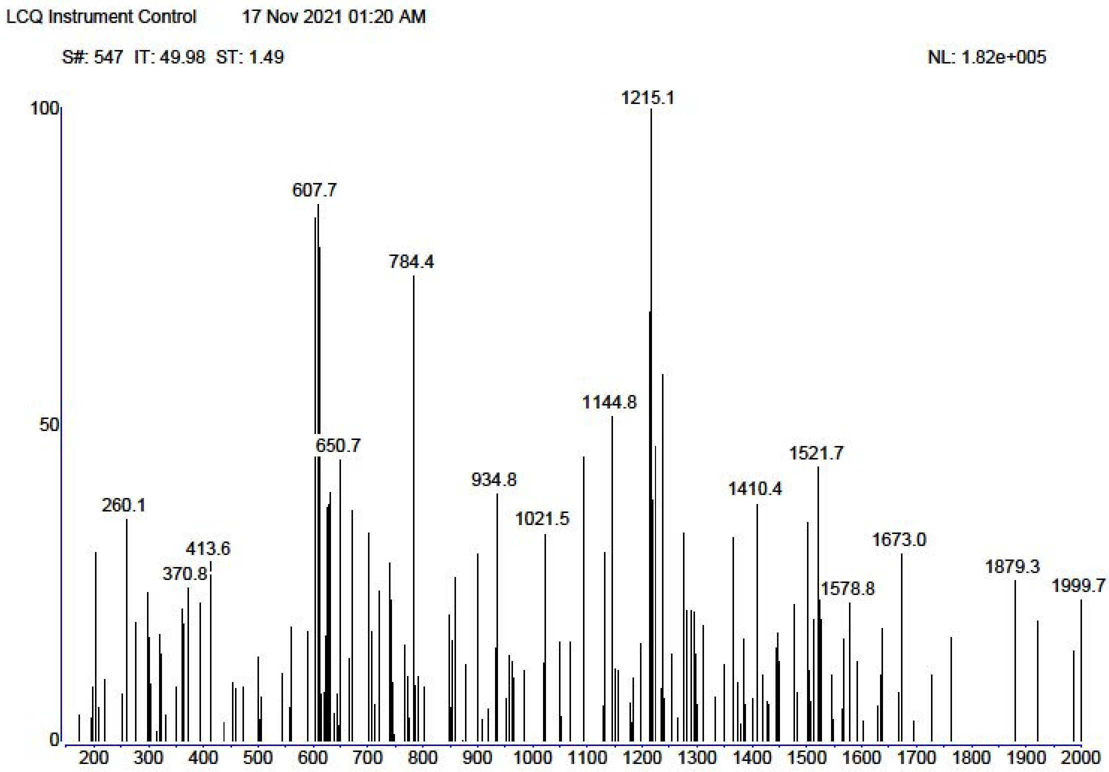

LCQ Instrument Control 17 Nov 2021 01:20 AM
S#: 547 IT: 49.98 ST: 1.49
NL: 1.82e+005
100
50
0
1215.1
607.7
784.4
1144.8
650.7
1521.7
934.8
1410.4
260.1
1021.5
413.6
1673.0
370.8
1879.3
1578.8
1999.7
200 300 400 500 600 700 800 900 1000 1100 1200 1300 1400 1500 1600 1700 1800 1900 2000

TXGS-5

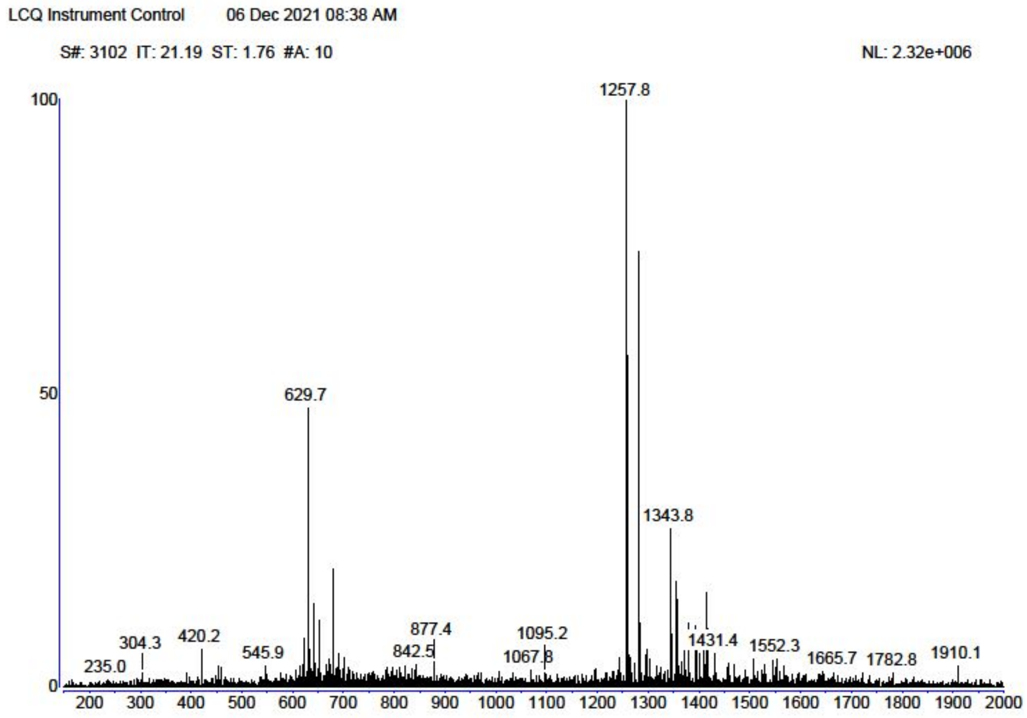

LCQ Instrument Control 06 Dec 2021 08:38 AM
S#: 3102 IT: 21.19 ST: 1.76 #A: 10
NL: 2.32e+006
100
50
0
1257.8
629.7
1343.8
877.4
1095.2
304.3
420.2
1431.4
1552.3
545.9
842.5
1067.8
1665.7
1782.8
1910.1
235.0
200 300 400 500 600 700 800 900 1000 1100 1200 1300 1400 1500 1600 1700 1800 1900 2000

TXGS-6

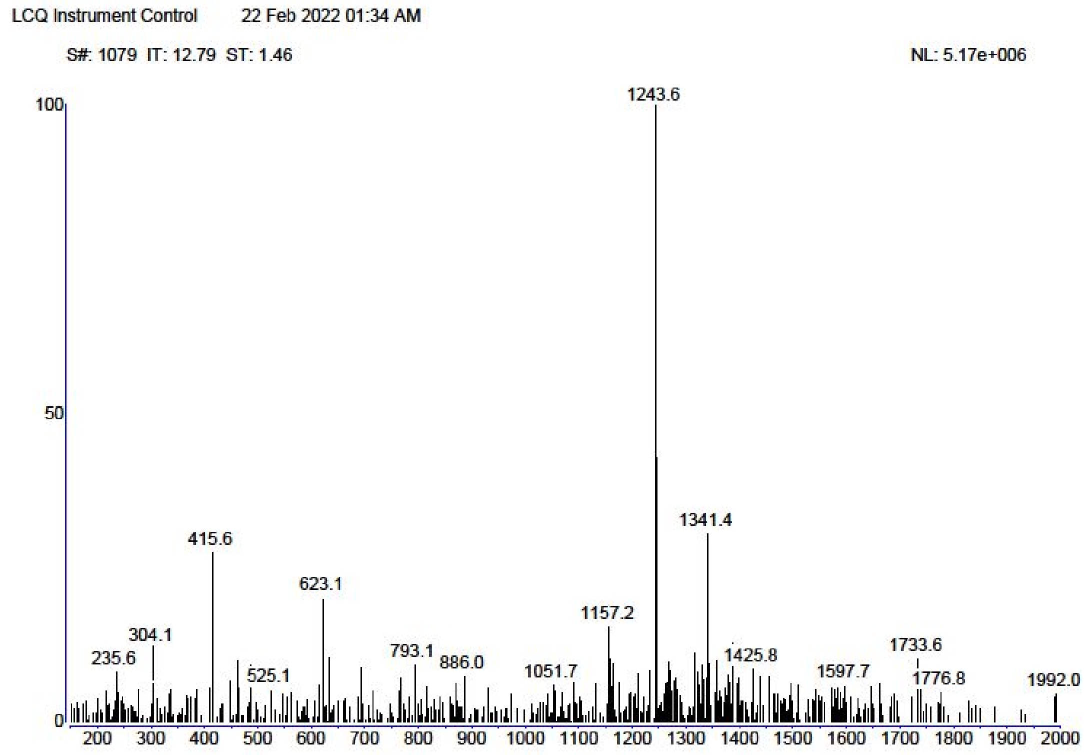
LCQ Instrument Control 22 Feb 2022 01:34 AM
S#: 1079 IT: 12.79 ST: 1.46
NL: 5.17e+006
1243.6
1341.4
415.6
623.1
1157.2
304.1
235.6
525.1
793.1
886.0
1051.7
1425.8
1597.7
1733.6
1776.8
1992.0
100
50
0
200 300 400 500 600 700 800 900 1000 1100 1200 1300 1400 1500 1600 1700 1800 1900 2000

TXGS-7

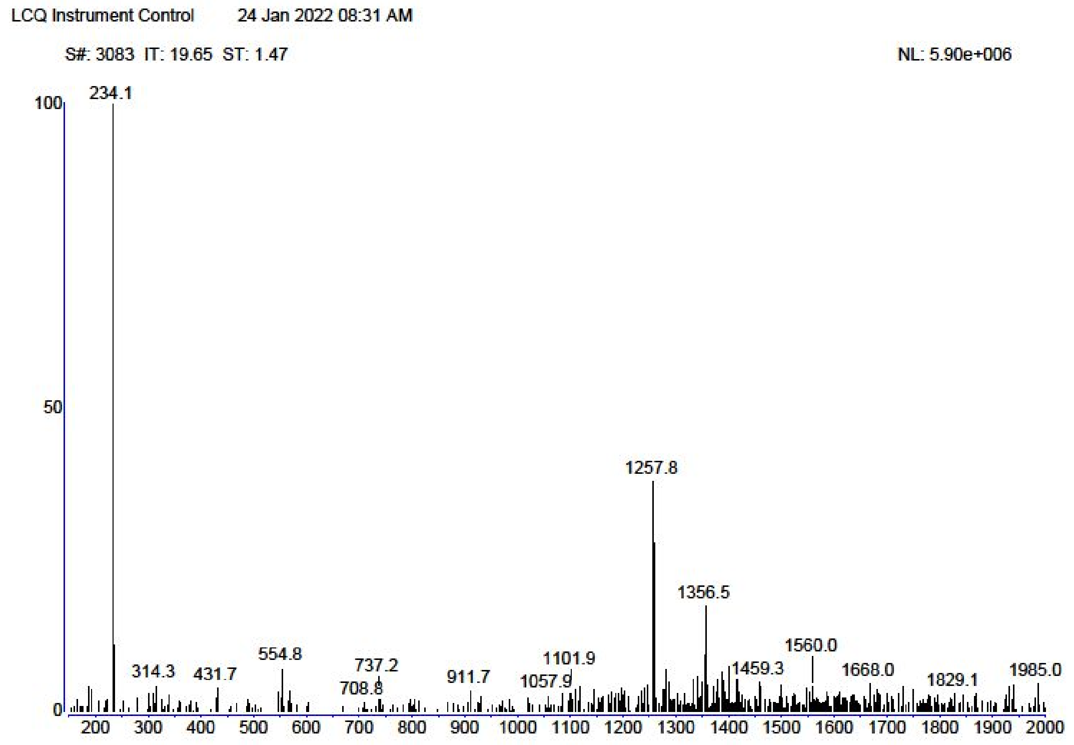
LCQ Instrument Control 24 Jan 2022 08:31 AM
S#: 3083 IT: 19.65 ST: 1.47
NL: 5.90e+006
234.1
1257.8
1356.5
314.3
431.7
554.8
737.2
708.8
911.7
1101.9
1057.9
1459.3
1560.0
1668.0
1829.1
1985.0
100
50
0
200 300 400 500 600 700 800 900 1000 1100 1200 1300 1400 1500 1600 1700 1800 1900 2000

TXGS-8

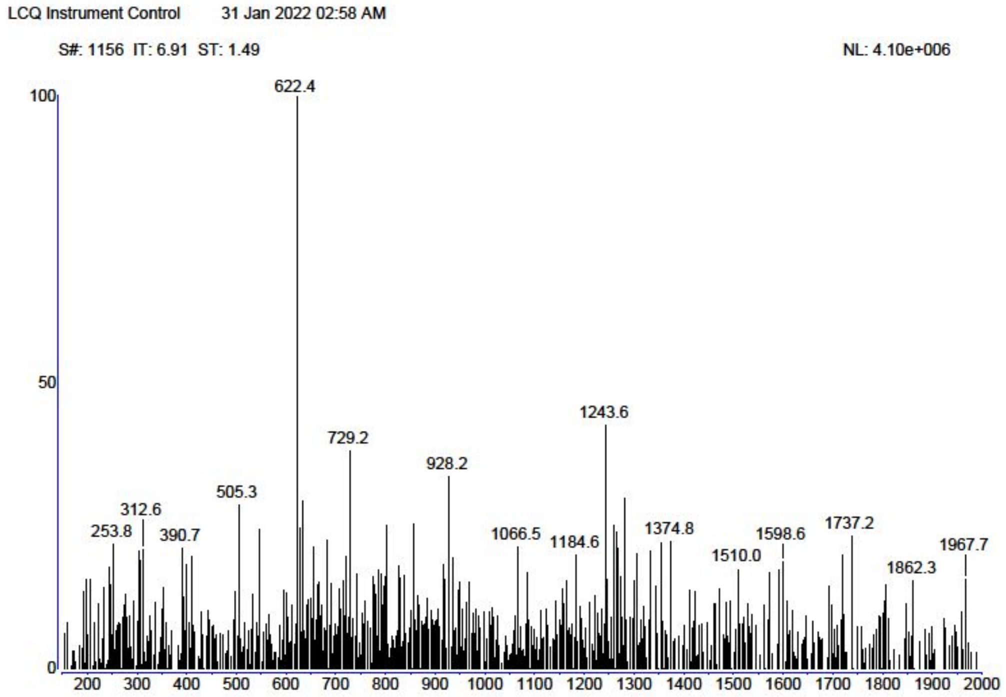
LCQ Instrument Control 31 Jan 2022 02:58 AM
S#: 1156 IT: 6.91 ST: 1.49
NL: 4.10e+006
100
50
0
622.4
729.2
928.2
1243.6
505.3
312.6
253.8
390.7
1066.5
1184.6
1374.8
1510.0
1598.6
1737.2
1862.3
1967.7
200 300 400 500 600 700 800 900 1000 1100 1200 1300 1400 1500 1600 1700 1800 1900 2000
